# Verdickte und sezernierende Nabelschnur bei einem Neugeborenen

**DOI:** 10.1007/s00104-025-02418-5

**Published:** 2025-11-27

**Authors:** Christoph Pensko, Thomas Meyer

**Affiliations:** Abteilung für Kinder- und Jugendchirurgie – Kinderurologie und Kindertraumatologie, Klinik und Poliklinik für Allgemein‑, Viszeral‑, Transplantations‑, Gefäß- und Kinderchirurgie, Zentrum Operative Medizin (ZOM), Universitätsklinikum Würzburg, Oberdürrbacher Str. 6, 97080 Würzburg, Deutschland

## Anamnese

Im vorliegenden Fall handelt es sich um ein eutrophes, reifes Neugeborenes der 37 + 0 SSW einer 1. Gravida, 1. Para mit einem Geburtsgewicht von 2580 g. Erstmals wurde im Rahmen einer sonographischen Untersuchung im Gestationsalter von 16 Wochen + 3 der Verdacht auf eine Omphalozele mit Darmschlingen als Inhalt geäußert. Eine Chromosomenanalyse nach Amniozentese ergab einen unauffälligen Befund. Das Kind wurde per primärer Sectio entbunden, der Apgar-Test ergab 9/10/10.

## Klinischer Befund

**Abb. 1:**
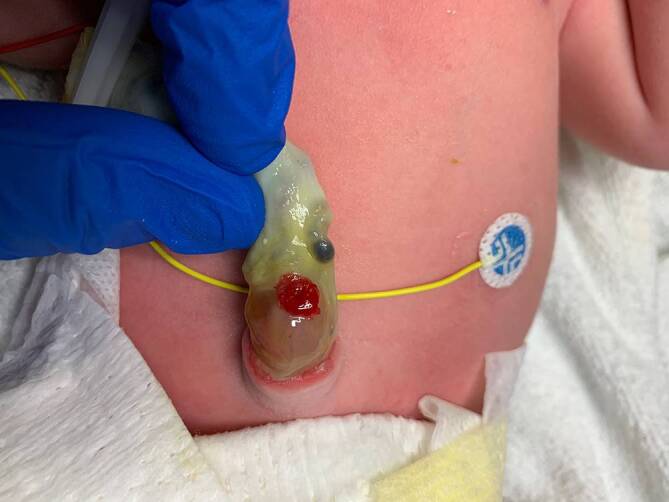
Postnataler Befund des verdickten und sezernierenden Nabels mit einem sichtbaren „Ostium“ im Bereich der Nabelschnur

In der ersten körperlichen postnatalen Inspektion wurde eine „kleine Omphalozele“ mit vorhandener enteraler Fistel beschrieben (Abb. 1). Hierüber entleerten sich Mekonium und im Verlauf auch Übergangsstuhl. Nebenbefundlich bestand ein persistierendes Foramen ovale mit kleinem Vorhofseptumaneurysma, die übrigen kardiovaskulären Strukturen waren unauffällig.

## Wie lautet Ihre Diagnose?

## Weiteres Procedere

**Abb. 2:**
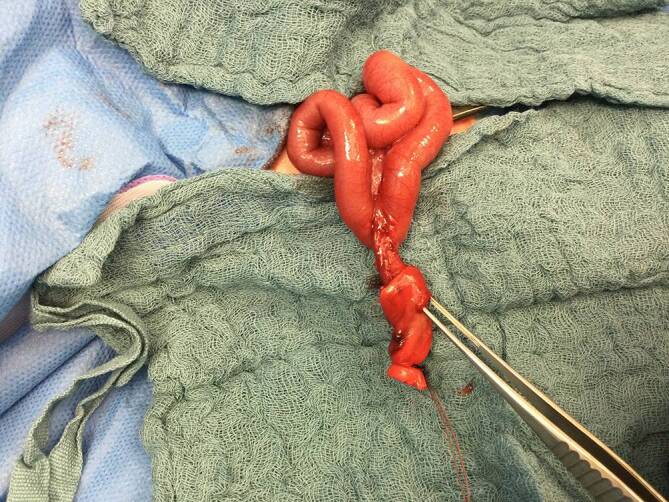
Intraoperativer Befund der Nabelschnurhernie mit dem in die Nabelschnur „perforierten“ persistierenden Ductus omphaloentericus (Pinzette liegt im äußeren Ostium des Ductus omphaloentericus an der Nabelschnur)

Nach einer unauffälligen postnatalen Adaptation wurde das Neugeborene noch am 1. Lebenstag zur weiteren Therapie in die Kinderchirurgie verlegt. Intraoperativ zeigte sich ein persistierender Ductus omphaloentericus mit „Perforation“ im Bereich der auffällig verdickten Nabelschnur (Abb. 2). Es erfolgten eine Segmentresektion und nachfolgende Passagerekonstruktion mittels terminoterminaler Ileoileostomie. Anschließend erfolgte die Nabelrekonstruktion mittels einer Tabaksbeutelnaht. Hierunter zeigte sich ein gutes kosmetisches Ergebnis. Ab dem 2. Post-OP-Tag wurde das Kind problemlos enteral kostaufgebaut.

## Histologie

Die pathologisch-anatomische Begutachtung zeigte ein Dünndarmresektat mit einem 3,6 cm langen persistierenden Ductus omphaloentericus, unmittelbar an die Nabelschnur angrenzend.

## Diskussion

Eine Nabelschnurhernie („hernia into the cord“) ist eine seltene Pathologie mit einem Aufkommen von 1:5000 [1] und wird neben der Omphalozele, der Gastroschisis [2] und den kongenitalen kindlichen Nabelhernien zu den ventralen Bauchwanddefekten gezählt [3, 4]. Häufig bleibt sie unerkannt [1, 5] oder wird fälschlicherweise für eine kleine Omphalozele gehalten [5, 6]. Die Nabelschnurhernie kann unterschiedliche Ausmaße annehmen. Klinisch imponiert sie am häufigsten als kleine Schwellung im Bereich des Nabelschnuransatzes bei einem asymptomatischen Neugeborenen [3]. In der Nabelschnurhernie können prolabierte Anteile des Ileums, selten aber auch ein Überbleibsel des Ductus omphaloentericus gefunden werden. Der Entwicklungszeitpunkt dieser Pathologie findet etwa in der 10. bis 12. Gestationswoche statt. Ursächlich ist am ehesten eine unvollständige Rückverlagerung der Abdominalorgane aus dem Coelom [5, 6]. Der Bauchnabel, die Nabelschnur sowie die Bauchmuskulatur sind hierbei physiologisch angelegt (s. auch Tab. 1).

**Tab. 1:** Differenzialdiagnose der ventralen Bauchdeckendefekte im Kindesalter. (Mod. nach [5])

	Nabelschnurhernie	Omphalozele	Gastroschisis	Nabelhernie
*Ätiologie*	Persistenz einer physiologischen Nabelherniation über die 10.–12. SSW hinaus	Fehlender Verschluss der ventralen Bauchwand	Unklare Pathologie, Verdacht auf Verschluss der A. omphalomesenterica, abnormale Rückbildung der rechten Umbilikalvene	Verzögerter Verschluss des Nabelrings
*Inzidenz*	1:5000	1:4000	1:2000	Sehr häufig
*Genetik*	Sporadisch	Meist sporadisch	Meist sporadisch	Sporadisch
*Assoziierte Fehlbildungen*	Seltene Fallberichte von assoziierten Darmanomalien	50–70 % der Patienten haben weitere Fehlbildungen	In 8–10 % der Fällt weitere „große“ Anomalien, in 1–3 % der Fälle zusätzliche Herzfehlbildungen	Selten
*Abnormer Karyotyp*	Kein Zusammenhang mit abnormalem Karyotyp	In 30–40 %	Kein Zusammenhang mit abnormalem Karyotyp	Kein Zusammenhang mit abnormalem Karyotyp
*Prognose*	Gut	Abhängig von assoziierten Fehlbildungen und Karyotyp	Gute Prognose, aber in 25 % Darmkomplikationen	Sehr gute Prognose

Bei Geburt ist das wichtigste diagnostische Kriterium die Morphologie des Nabelschnuransatzes, welcher physiologisch an einem intakten Nabelring anliegen sollte [1, 4]. In der häufigsten Zahl der Fälle erfolgt die chirurgische Versorgung der „hernia into the cord“ innerhalb der ersten 24 Lebensstunden, insbesondere im Falle einer bestehenden Symptomatik [1, 3, 5–9]. Kleine Hernien können sich aber auch ohne Operation reponieren und nachfolgend epithelisieren. Bei einer Nabelschnurhernie ist die Nabelschnur physiologisch an einem ausgebildeten Nabelring angelegt [1, 10–12]. Zudem besteht eine intakte Bauchwand mit lediglich einem lokalen Fasziendefekt. Der Bruchsack besteht aus äußerem Amnion sowie innerem Peritonealblatt [6, 11]. Im Gegensatz zur Nabelhernie, bei der Darmschlingen oder peritoneales Fett aus dem Bauchraum durch einen Fasziendefekt an der verschlossenen Bauchwand prolabieren, hat bei einer Nabelschnurhernie nur eine unvollständige Rückverlagerung der Darmschlingen stattgefunden [13]. Eine Assoziation mit chromosomalen Abnormitäten ist nicht bekannt [14], jedoch kann eine Assoziation mit weiteren gastrointestinalen Malformationen bestehen, wie z. B. einer Malrotation oder einem persistierenden Ductus omphaloentericus bzw. einem Meckel-Divertikel [3, 5–8].

**Diagnose:** Nabelschnurhernie mit einem „perforierten“ persistierenden Ductus omphaloentericus

Das Meckel-Divertikel ist die häufigste angeborene Fehlbildung des Gastrointestinaltrakts beim Menschen, etwa 2 % der Bevölkerung sind davon betroffen [9]. Während der fetalen Entwicklung ist der Dottersack mit dem primitiven Darm über den Ductus omphaloentericus verbunden. Bei unvollständiger Rückbildung kann es zu den verschiedenen Varianten des Meckel-Divertikels kommen [9].

## Fazit für die Praxis

Letztendlich ist die „hernia into the cord“ eine gutartige und isolierte Erkrankung, welche aber in Einzelfällen mit weiteren angeborenen Fehlbildungen, wie z. B. einem persistierenden Ductus omphaloentericus, einhergehen kann. Nach chirurgischer Versorgung hat die Nabelschnurhernie eine gute Prognose. Eine Differenzierung zwischen Omphalozele und Nabelschnurhernie als unterschiedliche eigenständige Entitäten ist sowohl aufgrund der unterschiedlichen Prognosen unter Berücksichtigung der assoziierten Fehlbildungen als auch aufgrund der Pathophysiologie klinisch relevant.
